# Clinical Outcomes of Coronavirus Disease 2019 in People Living With Human Immunodeficiency Virus in South Korea: A Nationwide Population‐Based Cohort Study

**DOI:** 10.1111/irv.13337

**Published:** 2024-06-10

**Authors:** Jeong Yeon Kim, Yujin Jeong, Hyonggin An, Jin Woong Suh, Jang Wook Sohn, Young Kyung Yoon

**Affiliations:** ^1^ Division of Infectious Diseases, Department of Internal Medicine Korea University College of Medicine Seoul Republic of Korea; ^2^ Department of Biostatistics Korea University College of Medicine Seoul Republic of Korea

**Keywords:** cohort studies, coronavirus disease 2019, epidemiology, human immunodeficiency virus, SARS‐CoV‐2, treatment outcome

## Abstract

**Background:**

We aimed to compare the epidemiological and clinical characteristics of coronavirus disease 2019 (COVID‐19) in people living with human immunodeficiency virus (HIV) (PLWH) with those in people living without HIV (PLWoH).

**Methods:**

This nationwide descriptive epidemiological study was conducted in South Korea between January 2020 and February 2022. The National Health Insurance claim data, comprising the data of the entire Korean population, were collected through the Health Insurance Review and Assessment Service.

**Results:**

Among 3,653,808 individuals who were diagnosed with COVID‐19, 1311 (0.04%) were PLWH. All PLWH received antiretroviral therapy, and 26.47% had more than one underlying disease other than HIV infection. The overall in‐hospital mortality rates of PLWH and PLWoH were 0.76% and 0.25%, respectively (*P* = 0.002). According to the Cox proportional hazard model, no significant difference was observed in the in‐hospital mortality rate (hazard ratio [HR]: 1.80, 95% confidence interval [CI]: 0.70–4.67) between the PLWH and PLWoH. However, progression to severe or critical COVID‐19 was more common in PLWH (HR: 2.70, 95% CI: 1.37–5.33). In PLWH diagnosed with COVID‐19, a multivariable Cox regression analysis found old age (≥ 60 years) (HR: 6.9, 95% CI: 2.57–18.56) and diabetes mellitus (HR: 5.13, 95% CI: 2.02–13.00) as the independent risk factors for severe or critical COVID‐19.

**Conclusions:**

PLWH had a significantly higher risk of developing severe or critical COVID‐19 compared with PLWoH. Our findings suggest the need for implementing tailored strategies to decrease the impact of COVID‐19 on PLWH.

## 1 Introduction

The coronavirus disease 2019 (COVID‐19) pandemic, caused by the severe acute respiratory syndrome coronavirus 2 (SARS‐CoV‐2), continues to impose a substantial global burden. Contrary to expectations concerning its eradication, SARS‐CoV‐2 has become an endemic pathogen with sporadic epidemic peaks [1]. Recently, the COVID‐19 pandemic has overshadowed other infectious diseases; managing patients with coinfections remains a significant concern.

People living with human immunodeficiency virus (HIV) (PLWH), particularly those with viremia and immunosuppression, are at increased risk of COVID‐19‐related complications [2]. However, the exact interaction between SARS‐CoV‐2 and HIV infection remains unclear. The Republic of Korea (ROK), a low HIV prevalence country, reported 15,880 HIV infections with a prevalence of less than 0.1% in 2023. However, the number of a new HIV diagnoses has risen since the early 2000s: 244 in 2000, 837 in 2010, and 1066 in 2022 [3].

A higher prevalence of underlying comorbidities and persisting immunodeficiency could increase COVID‐19 severity in PLWH compared with the people living without HIV (PLWoH) [4, 5]. A cohort study in South Africa found association between HIV infection and higher mortality, particularly among individuals not receiving antiretroviral therapy (ART) [6]. Conversely, immunosuppression might limit COVID‐19's hyperinflammatory response [7]. Early in the pandemic, hospitalized virologically suppressed PLWH on ART had similar outcomes to other hospitalized PLWoH [8]. Some ART regimens that inhibit SARS‐CoV‐2 may also reduce disease severity [9, 10].

In summary, the available clinical data provide contradictory findings regarding the potential impact of HIV on the severity of COVID‐19 [6, 8, 11, 12]. These inconsistent findings may have been influenced by the small sample size, differences in vaccine coverage, overall differences in the care type received, or the study population used.

Therefore, this population‐based nationwide study is aimed at investigating the epidemiology of COVID‐19 in PLWH, comparing the clinical severity of COVID‐19 between the PLWH and PLWoH, and assessing the risk of severe or critical illness in this subpopulation.

## 2 Materials and Methods

### 2.1 Study Design

This nationwide, population‐based, cohort study was conducted between January 2020 and February 2022 in the ROK. In patients with multiple episodes of COVID‐19, only the data from the first episode were included in the analysis to avoid bias from the duplicate.

The primary study outcome was to compare the COVID‐19‐related hospitalization and in‐hospital mortality rates between the PLWH and PLWoH. The secondary outcomes included the composite outcomes of severe or critical COVID‐19 requiring noninvasive or invasive ventilation, high‐flow oxygen therapy, and extracorporeal membrane oxygenation (ECMO). Additionally, the association between ART treatment against COVID‐19 and severity among PLWH was evaluated.

### 2.2 Study Setting

As of December 2022, the ROK's population had reached 51,439,038 individuals, of whom 15,196 were PLWH [13, 14]. In the ROK, the first case of COVID‐19 was reported on January 20, 2020, and the first vaccine was administered on February 26, 2021. By February 28, 2022, 87.4%, 86.4%, and 61.1% of the population had been vaccinated against COVID‐19 once, twice, and thrice, respectively [15].

In the ROK, healthcare is provided under the National Health Insurance (NHI) program, which covers the entire population. The Ministry of Health and Welfare oversees this comprehensive social insurance program. Healthcare providers submit claims through the Health Insurance Review and Assessment Service (HIRA) web portal.

The ROK implemented a proactive policy for COVID‐19 testing during the study period. As part of this approach, facilities dedicated for COVID‐19 testing were established in public health centers, allowing individuals to undergo testing for free. The ROK implemented proactive COVID‐19 testing policies during the study period, establishing facilities for free testing in public health centers. In the ROK healthcare system, ART and COVID‐19 vaccination are comprehensively funded through public taxes. All COVID‐19 patients were mandatorily admitted to residential treatment centers or hospital under government supervision until August 2021. Since March 2020, residential treatment centers were established for patients with asymptomatic or mild cases, owing to the limited availability of hospital beds.

### 2.3 Data Sources

Data for this study were collected from the NHI claim database, accessed via the HIRA's web‐based Medical Claim Portal Service. These data included demographic details, insurance types, healthcare facility codes, care types, treatment dates, diagnosis codes assigned based on the International Classification of Diseases 10th Revision (ICD‐10), medical procedures, patient outcomes, and medications prescribed.

### 2.4 Definitions

The study included patients from ROK with confirmed COVID‐19 between January 2020 and February 2022. Diagnostic codes were assigned based on the Seventh Revision of the Korean Classification of Diseases (KCD‐7), a modified version of the ICD‐10. Codes for COVID‐19 were assigned after confirmation via real‐time reverse transcription‐polymerase chain reaction assays (Table S1). The index date was the COVID‐19 diagnosis date. Additionally, PLWH were identified using the KCD‐7/ICD‐10 codes (Table S1). All patients were monitored from January 2020 to February 2022. The study period was determined based on the extent of the data available from the HIRA at the beginning of the analysis.

The COVID‐19 treatment (Table S2), ART (Table S3), and vasopressors (Table S4) were identified using the Anatomical Therapeutic Chemical codes and HIRA general name codes. Medications for COVID‐19 treatment were considered relevant if prescribed within 2 weeks from the index date to prevent bias. The ART medications prescribed before the index date were deemed relevant for inclusion.

Data on hospital and intensive care unit (ICU) admissions were analyzed within 2 weeks after the index date. The hospitalized patients were evaluated based on the vasopressor prescription status and procedure codes identifying those who received conventional oxygen therapy, high‐flow oxygen therapy, mechanical ventilation, ECMO, and renal replacement therapy (Table S4). Disease severity was categorized using the National Institute of Allergy and Infectious Diseases Ordinal Scale for COVID‐19 severity, which classifies hospitalization as Scale 3, oxygen requirement as Scale 5, and severe or critical COVID‐19 as Scale 6 or higher [16]. The underlying comorbidities were determined from claims within 6 months before the index date and evaluated using the Charlson Comorbidity Index (CCI), which predicted the 10‐year mortality in patients with various comorbid conditions (Table S5) [17].

### 2.5 Statistical Analysis

The baseline characteristics were expressed as the mean ± standard deviations or median with interquartile ranges (IQRs) for continuous variables and frequency for categorical variables. The baseline characteristics between PLWH and PLWoH were compared using the independent *t*‐test, chi‐square test, or Fisher's exact test as appropriate.

To account for potential selection bias and maintain a balance in the confounding factors between the two groups, we calculated the inverse probability of treatment weights (IPTWs) for each individual in the cohort. Age, sex, diabetes mellitus, chronic pulmonary disease, and chronic liver disease were used as covariates for the calculation of weights following the stabilized weight method.

A Cox proportional hazard model adjusted with IPTW was used to estimate the hazard ratio (HR) of COVID‐19 severity according to HIV status. Conversely, an IPTW‐adjusted logistic regression analysis was used to compare the COVID‐19 severity according to HIV status, enabling the calculation of the odds ratio (ORs). Within the PLWH group, the estimated HR and the corresponding 95% confidence interval (CI) of COVID‐19 outcomes according to the ART prescribed were compared using a Cox proportional hazard model adjusted for age and sex. To assess the risk factors associated with the clinical outcomes of COVID‐19 in total patients and the PLWH, a multivariable Cox regression analysis using the IPTW was conducted. A *P*‐value of less than 0.05 was considered significant. All statistical analyses were performed using the SAS software (version 9.4; SAS Institute, Cary, NC, USA).

## 3 Results

### 3.1 Characteristics of the Study Population

**FIGURE 1:**
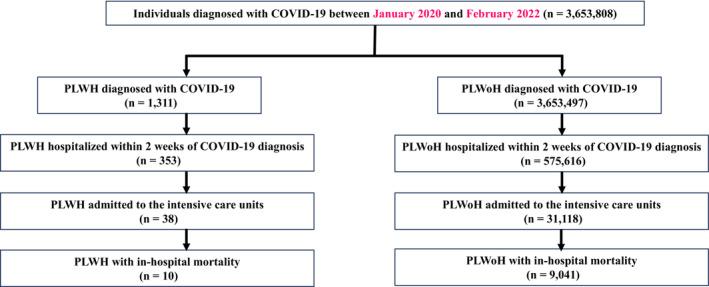
Flow diagram showing the participant selection process. PLWH, people living with human immunodeficiency virus; PLWoH, people living without human immunodeficiency virus.

The database contains claim data from 3,653,808 individuals who were diagnosed with COVID‐19 and from hospitals that issued claims to the HIRA between January 2020 and February 2022. The age distribution of the patients is presented in Table 1. Approximately 46.3% of the cohort were men. Among them, 575,969 (15.76%) required hospitalization, with 31,156 (0.85%) being admitted to the ICU and 9051 (0.25%) died (Figure 1). Among the 3,653,808 individuals with COVID‐19, 1311 (0.04%) were PLWH. The overall incidence rates of COVID‐19 were 7.2% and 8.6% in the PLWoH and PLWH (approximately 51,000,000 people and 15,880 PLWH were living in the ROK), respectively. All PLWH received ART, and 26.47% had more than one underlying disease other than an HIV infection.

**TABLE 1:** Comparison of baseline and clinical characteristics of COVID‐19 between people living with HIV and people living without HIV in the Republic of Korea.

	Total (*n* = 3,653,808)	PLWoH (*n* = 3,652,497)	PLWH (*n* = 1311)	***P*‐value**
Male, *n* (%)	1,691,002 (46.3)	1,689,841 (46.3)	1161 (88.6)	< 0.001
Age (years), *n* (%)				< 0.001
< 20	905,385 (24.8)	905,368 (24.8)	17 (1.3)	
20–29	558,448 (15.8)	558,189 (15.3)	259 (19.8)	
30–39	575,748 (15.8)	575,370 (15.8)	378 (28.83)	
40–49	581,293 (15.9)	581,049 (15.9)	244 (18.6)	
50–59	431,348 (11.8)	431,170 (11.8)	178 (13.6)	
60–69	360,587 (9.9)	360,439 (9.9)	148 (11.3)	
70–79	153,271 (4.2)	153,207 (4.2)	64 (4.9)	
≥80	87,728 (2.4)	87,705 (2.4)	23 (1.8)	
Comorbidities, *n* (%)				
Diabetes mellitus	291,015 (8.0)	290,866 (8.0)	149 (11.4)	< 0.001
Congestive heart failure	15,921 (0.44)	15,913 (0.44)	8 (0.61)	0.337
Cerebrovascular diseases	4511 (0.12)	4507 (0.12)	4 (0.31)	0.081
Chronic pulmonary diseases	249,163 (6.82)	249,034 (6.82)	129 (9.84)	< 0.001
Chronic liver diseases	241,340 (6.61)	241,208 (6.6)	132 (10.07)	< 0.001
Chronic kidney diseases	18,810 (0.51)	18,797 (0.51)	13 (0.99)	0.016
Malignancy	37,859 (1.04)	37,840 (1.04)	19 (1.45)	0.140
Charlson Comorbidity Index				
Mean ± standard deviation, range (min, max)	0 ± 1 (0, 7)	0 ± 1 (0, 7)	0 ± 1 (0, 5)	< 0.001
Ranges, *n* (%)				< 0.001
0–2	3,629,387 (99.3)	3,628,096 (99.3)	1291 (98.5)	
3–5	24,410 (0. 7)	24,390 (0. 7)	20 (1.5)	
≥6	11 (0)	11 (0)	0 (0)	
COVID‐19 treatment, *n* (%)				
Hospitalization	575,969 (15.8)	575,616 (15.8)	353 (26.9)	< 0.001
ICU admission	31,156 (0.85)	31,118 (0.9)	38 (2.9)	< 0.001
Corticosteroids	373,497 (10.2)	373,318 (10.2)	179 (13.7)	< 0.001
Dexamethasone	122,923 (3.4)	122,849 (3.4)	74 (5.6)	< 0.001
Methylprednisolone	125,339 (5.4)	195,254 (5.4)	85 (6.5)	0.067
Prednisolone	98,813 (2.7)	98,760 (2.7)	53 (4.0)	0.003
Hydrocortisone	4623 (0.1)	4622 (0.1)	1 (0.08)	1
Anticoagulants	40,576 (1.1)	40,532 (1.11)	44 (3.4)	< 0.0001
Heparin	10,693 (0.3)	10,674 (0.29)	19 (1.5)	< 0.0001
Dalteparin	374 (0.01)	373 (0.01)	1 (0.08)	0.1256
Enoxaparin	33,586 (0.9)	33,558 (0.92)	28 (2.1)	< 0.0001
Tocilizumab	198 (0.01)	198 (0.01)	0	1
Baricitinib	2218 (0.06)	2213 (0.06)	5 (0.4)	0.0014

Abbreviations: COVID‐19, coronavirus disease 2019; ICU, intensive care units; Max, maximum; Min, minimum; PLWH, people living with HIV; PLWoH, people living without HIV.

Table 1 presents the baseline characteristics of patients with COVID‐19 based on their HIV status. The proportion of male patients in the HIV group was higher than that in PLWoH. Moreover, the age distribution of patients with COVID‐19 differed between the two groups. In terms of comorbidities, the PLWH exhibited a higher prevalence of diabetes, chronic pulmonary disease, chronic liver disease, and chronic renal diseases compared with the PLWoH (Table 1). Approximately 26.47% and 18.59% of the individuals included in the PLWH and PLWoH groups had more than one underlying disease other than HIV infection, respectively (*P* = 0.001). The CCI of ≥ 3 was more prevalent in PLWH than in PLWoH (Table 1). PLWH were more likely to be prescribed glucocorticosteroids (*P* < 0.001) or anticoagulants (*P* < 0.001) within 2 weeks from COVID‐19 diagnosis than PLWoH (Table 1).

### 3.2 Outcomes of COVID‐19 According to HIV Status

The overall in‐hospital mortality rates of the PLWH and PLWoH were 0.76% and 0.25%, respectively (*P* = 0.002). The nationwide COVID‐19 hospitalization rates were 15.8% in the PLWoH (approximately 51,000,000 people were living in the ROK in 2022) and 26.9% among the PLWH (approximately 15,880 in the ROK in 2022; *P* < 0.001). Moreover, 5.7% and 2.2% of the PLWH and PLWoH, respectively, required supplemental oxygen (*P* < 0.001). Notably, 8% and 0.6% of the PLWH and PLWoH, respectively, developed severe or critical COVID‐19 (*P* < 0.001). The HRs for hospitalization and in‐hospital mortality in the PLWH were 1.07 (95% CI: 0.99–1.17) and 2.16 (95% CI: 0.82–5.70), respectively, and were not significantly different from those in the PLWoH (*P* = 0.100 and *P* = 0.118, respectively).

In Table 2, the ORs for various treatments required during hospitalization are summarized using logistic regression analysis adjusted for IPTW. The odds of overall oxygen therapy (OR: 2.33, 95% CI: 1.80–3.03, *P* < 0.001) or vasopressor use (OR: 3.18, 95% CI: 2.00–5.06, *P* < 0.001) were higher in the PLWH than in the PLWoH. Similarly, the odds of severe or critical COVID‐19 (OR: 3.43, 95% CI: 2.28–5.16, *P* < 0.001) were higher in the PLWH than in the PLWoH (Table 2).

**TABLE 2:** Comparison of clinical severity according to HIV status in the hospitalized patients using logistic regression analysis adjusted with inverse probability weighting.

Variables	Event, *n* (%)	OR	95% CI of OR		*P*‐value
Oxygen inhalation using nasal cannula or mask					
HIV	70 (0)	2.17	1.66	2.85	< 0.001
Non‐HIV	79,820 (2.2)	Reference			
High‐flow nasal cannula					
HIV	23 (0)	4.31	2.85	6.50	< 0.001
Non‐HIV	16,093 (0.4)	Reference			
Mechanical ventilation					
HIV	9 (0)	4.33	2.43	7.74	< 0.001
Non‐HIV	7454 (0.2)	Reference			
Extracorporeal membrane oxygenation					
HIV	3 (0)	17.16	6.75	43.63	< 0.001
Non‐HIV	679 (0.02)	Reference			
Overall oxygen therapy					
HIV	75 (0)	2.33	1.80	3.03	< 0.001
Non‐HIV	81,884 (2.2)	Reference			
Vasopressor					
HIV	20 (0)	3.18	2.00	5.06	< 0.001
Non‐HIV	16,178 (0.4)	Reference			
Severe and critical COVID‐19					
HIV	24 (0)	3.43	2.28	5.16	< 0.001
Non‐HIV	20,701 (0.6)	Reference			

Abbreviations: CI, confidence interval; COVID‐19, coronavirus disease‐2019; HIV, human immunodeficiency virus; ORs, odds ratios.

### 3.3 COVID‐19 Outcomes by Tenofovir Use Among the PLWH

All PLWH received ART. The most common ART regimen used was integrase strand transfer inhibitor (INSTI) plus two nucleotide reverse transcriptase inhibitors (NRTIs) (70.94%), followed by INSTI plus NRTIs (14.95%), non‐NRTIs (NNRTIs) plus two NRTIs (2.52%), protease inhibitors plus two NRTIs (0.99%), and others (24.3%). Among the PLWH, 53.1% received tenofovir alafenamide (TAF)/emtricitabine (FTC), 25.7% received abacavir (ABC)/lamivudine (3TC), and 2.7% received tenofovir disoproxil fumarate (TDF)/FTC.

The estimated HRs (95% CIs) of ICU admission and severe or critical COVID‐19 according to the type of ART regimen used in the PLWH were analyzed using a Cox proportional hazard model adjusted with IPTW for age and sex (Table 3). The HRs were calculated with the reference being the combined group of all other regimens. The estimated HRs of ICU admission and severe or critical COVID‐19 in patients who received TAF/FTC were the lowest, accounting for 0.39 (95% CI: 0.19–0.81) and 0.30 (95% CI: 0.12–0.75), respectively (Table 3).

**TABLE 3:** Estimated hazard ratios of COVID‐19 outcomes based on the type of antiretroviral therapy regimens used in people living with HIV.^a^

	COVID‐19 hospitalization		ICU admission		Severe or critical COVID‐19		Death	
	No. of events	Hazard ratios (95% CI)	No. of events	Hazard ratios (95% CI)	No. of events	Hazard ratios (95% CI)	No. of events	Hazard ratios (95% CI)
TAF/FTC	169	1.24 (0.83–1.86)	12	**0.39 (0.19–0.81)**	7	**0.30 (0.12–0.75)**	3	0.30 (0.05–1.88)
TDF/FTC	10	1.27 (0.61–2.65)	1	2.63 (0.31–22.23)	1	0.58 (0.06–5.42)	1	0.94 (0.06–15.87)
ABC/3TC	78	1.21 (0.82–1.77)	11	1.75 (0.67–4.63)	5	0.59 (0.20–1.77)	3	1.24 (0.27–5.74)
Other regimens	18	0.59 (0.31–1.13)	1	0.47 (0.06–3.46)	0	‐	0	‐

*Note:* Hazard ratios (HRs) are calculated with the reference being the combined group of all other regimens not listed in the same row (e.g., for TAF/FTC, the reference is TDF/FTC + ABC/3TC + other regimens). Bold emphasis indicates statistical significance.

Abbreviations: 3TC, lamivudine; ABC, abacavir; CI, confidence interval; COVID‐19, coronavirus disease‐2019; FTC, emtricitabine; ICU: intensive care unit; TAF, tenofovir alafenamide; TDF, tenofovir disoproxil fumarate.

^a^ Cox proportional hazard model adjusted with inverse probability of treatment weighting for age and sex.

### 3.4 Risk Factors Associated With the Clinical Outcomes in Patients With COVID‐19

In the patients diagnosed with COVID‐19, old age (≥ 60 years); male sex; a CCI value of ≥ 3; the presence of diabetes mellitus, chronic heart failure, cerebrovascular disease, chronic pulmonary disease, chronic kidney disease, and malignancy; and a history of organ transplant were independently associated with increased mortality (Table 4). However, being categorized as PLWH was not associated with in‐hospital mortality. Conversely, being categorized as PLWH was independently associated with severe or critical COVID‐19 along with old age (≥ 60 years); male sex; a CCI value of ≥ 3; and the presence of diabetes mellitus, chronic heart failure, cerebrovascular diseases, chronic pulmonary diseases, chronic liver diseases, chronic kidney diseases, malignancy, and rheumatic diseases (Table 4).

**TABLE 4:** Multivariable Cox regression analysis using the IPTW of the risk factors for COVID‐19 outcome in patients with COVID‐19.

	Severe or critical COVID‐19 (*n* = 20,725)		In‐hospital mortality (*n* = 9051)	
	Hazard ratios (95% CI)	*P*‐value	Hazard ratios (95% CI)	*P*‐value
Age ≥ 60 years	12.01 (11.58–12.46)	**< 0.001**	44.32 (40.75–48.21)	**< 0.001**
Male	1.55 (1.51–1.60)	**< 0.001**	1.27 (1.22–1.33)	**< 0.001**
CCI value of ≥ 3	0.63 (0.58–0.69)	**< 0.001**	0.70 (0.62–0.79)	**< 0.001**
PLWH	2.70 (1.37–5.33)	**0.004**	1.80 (0.70–4.67)	0.224
Diabetes mellitus	1.72 (1.66–1.78)	**< 0.001**	1.49 (1.42–1.56)	**< 0.001**
Chronic heart failure	4.12 (3.88–4.37)	**< 0.001**	1.96 (1.75–2.18)	**< 0.001**
Cerebrovascular diseases	2.08 (1.82–2.38)	**< 0.001**	2.04 (1.69–2.47)	**< 0.001**
Chronic pulmonary diseases	1.84 (1.78–1.91)	**< 0.001**	1.93 (1.83–2.04)	**< 0.001**
Chronic liver disease	1.07 (1.03–1.11)	**0.002**	0.92 (0.86–0.98)	**0.007**
Chronic kidney diseases	2.57 (2.38–2.77)	**< 0.001**	2.27 (2.03–2.54)	**< 0.001**
Malignancy	1.22 (1.12–1.32)	**< 0.001**	1.29 (1.16–1.45)	**< 0.001**
Rheumatic diseases	2.74 (1.52–4.95)	**< 0.001**	1.758 (0.580–5.321)	0.318
Organ transplantation	5.27 (0.78–35.74)	0.089	13.28 (1.78–100.27)	**0.012**

*Note:* Bold emphasis indicates statistical significance.

Abbreviations: CCI, Charlson Comorbidity Index; CI, confidence interval; COVID‐19, coronavirus disease 2019; HIV, human immunodeficiency virus; PLWH, people living with human immunodeficiency virus.

In the PLWH diagnosed with COVID‐19, old age (≥ 60 years) (HR: 6.9, 95% CI: 2.57–18.56, *P* < 0.001) and diabetes mellitus (HR: 5.13, 95% CI: 2.02–13.00, *P* < 0.001) were independently associated with severe or critical COVID‐19. However, treatment with the TAF‐containing regimen (HR: 0.518, 95% CI: 0.16–1.70, *P* = 0.277) or the TDF‐containing regimen (HR: 0.958, 95% CI: 0.05–18.45, *P* = 0.977) was not significantly associated with severe or critical COVID‐19.

## 4 Discussion

In this nationwide cohort study conducted in the ROK, the PLWH exhibited a 2.5‐fold greater risk of experiencing more severe COVID‐19 compared with the PLWoH. Compared with other ART regimens, TAF/FTC‐based regimens reduced the risk of developing severe COVID‐19 in the PLWH.

PLWH exhibited a 1.7‐fold higher incidence of COVID‐19 than the general population in the unadjusted analyses. This finding was similar to that of previous studies, which reported a higher susceptibility to COVID‐19 in the PLWH (up to 43%) than in the PLWoH [18, 19]. However, other studies reported conflicting results [20]. The susceptibility of PLWH to COVID‐19 can be attributed to the differences in age distribution and the prevalence of comorbidities among the study participants [21]. Notably, a recent meta‐analysis performed in 10 countries revealed no difference in the susceptibility to COVID‐19 between PLWH and PLWoH [22].

In our study, the PLWH exhibited hospitalization and in‐hospital mortality rates comparable to those of PLWoH, after adjusting for confounders including age, other comorbidities, and ART. These results were consistent with those of previous study [23]. Conversely, other studies yielded conflicting conclusions, with the adjusted HR for COVID‐19 mortality ranging from 1.69 to 2.59 in the PLWH compared with the PLWoH [24, 25]. Recent meta‐analyses revealed a higher risk of mortality among PLWH than among their HIV‐negative counterparts [26, 27]. This discordance may have resulted from the differences in the national response to the pandemic and population heterogeneity. In the ROK, until August 2021, all patients with COVID‐19 were required to stay in medical facilities including the residential treatment centers under government supervision. In other countries, the rates of COVID‐19‐related hospitalization among the PLWH ranged from 0.6% to 1.9% [24, 28]. Although the ROK has been considered as a low HIV prevalence country, its government has been thoroughly managing data on patients with COVID‐19 and PLWH based on health insurance claims. Thus, our study setting offered an advantage in investigating the association between COVID‐19 outcomes and HIV infection when HIV disease is well managed.

Our study demonstrated an increased severity of COVID‐19 in the PLWH compared with the PLWoH. However, the conclusion that they have increased risks of COVID‐19 hospitalization and mortality is not straightforward [21]. PLWH exhibited a significantly higher risk of requiring mechanical ventilation or vasopressors during COVID‐19 treatment. Consistent with our findings, a recent large‐scale study reported that PLWH exhibited 15% increased odds of developing severe or critical COVID‐19, after adjusting for other comorbidities [29]. The exaggerated host immune response increases the risk of a cytokine storm, potentially resulting in sepsis and acute respiratory distress syndrome [30], while the induction of strong protective immunity can facilitate viral replication control [31]. In PLWH, residual immunodeficiency and a higher prevalence of comorbidities could increase the risk of COVID‐19 exacerbation [32]. Conversely, immune dysfunction and certain ART regimens could potentially improve the COVID‐19 outcomes [33, 34].

A previous multicenter cohort study suggested that TDF‐based ART is associated with a decreased risk of hospitalization compared with other ART regimens [11, 34]. A small‐scale randomized trial also revealed an increased clearance of SARS‐CoV‐2 in the PLWH who received TDF/FTC compared with that in individuals who received the standard of care [35]. An in vitro study investigating molecular docking demonstrated that tenofovir strongly binds to ribonucleic acid (RNA)‐dependent RNA polymerase, providing a theoretical basis for its antiviral activity [10]. However, another cohort study showed no impact of tenofovir on outcomes [36]. The present study showed that TAF/FTC‐based regimens reduced the risk of developing severe COVID‐19, after adjusting for age and sex. However, in our multivariable analysis, the association of TAF/FTC with outcomes was not considered significant, which could be attributed to the small sample size in our cohort.

Our study identified that old age (≥ 60 years), male sex, a CCI value of ≥ 3, and medical comorbidities, including HIV infection, were significant risk factors associated with the progression to severe or critical COVID‐19 among PLWoH. A previous study demonstrated that noninfectious comorbidities such as diabetes mellitus, chronic pulmonary diseases, chronic liver diseases, and chronic kidney diseases are significantly more common in the PLWH than in the PLWoH, which is consistent with our findings [22, 37, 38]. In the PLWH, old age and diabetes mellitus were the significant risk factors associated with the progression to severe or critical COVID‐19, while ART regimens had no significant effect.

Our study has several limitations. First, when discussing the progression of COVID‐19 in PLWH, the clinical status of viremia control widely varies among individuals. However, this study failed to adjust for confounders such as viral burden and HIV disease progression. Furthermore, confounding variables such as the duration of ART and various socioeconomic factors were not included in the analysis. Nevertheless, all of the PLWH participating in this study were receiving ART and active treatment for COVID‐19. Notably, our study estimated the HR of COVID‐19 outcomes according to the type of ART regimens used in the PLWH while adjusting for age and sex. Second, we could not control for COVID‐19 vaccination status due to the absence of this information in the claim data. Hence, further studies are needed to evaluate the potential effect of vaccination against COVID‐19 on the clinical outcomes of PLWH. Lastly, the insurance claim records lacked clinical details and had potential for omission or misclassification of cases. However, this study included data on a large number of patients based on the nationwide population‐based electronic datasets.

In conclusion, our findings suggest a higher risk of severe COVID‐19 outcomes among the PLWH compared with the PLWoH. This offers valuable insights into the impact of COVID‐19 on PLWH and serves as a guide for establishing public health policies and guidelines for managing COVID‐19. Further global studies are necessary to understand the interplay between COVID‐19 and HIV infection.

## Author Contributions

**Jeong Yeon Kim:** formal analysis, writing–original draft. **Yujin Jeong:** formal analysis. **Hyonggin An:** formal analysis. **Jin Woong Suh:** resources. **Jang Wook Sohn:** resources. **Young Kyung Yoon:** conceptualization, supervision, writing–review and editing.

## Disclosure

The funding sources had no role in the study design, data collection, data analysis, decision to publish, or manuscript preparation.

## Ethics Statement

The study was approved by the institutional review board of Korea University Anam Hospital (approval no. 2022AN0272). The requirement for written informed consent was waived by the board, as all data used in the study were collected during the administration of routine care without the inclusion of any information that could potentially be used to identify individual patients.

## Conflicts of Interest

The authors declare no conflicts of interest.

### Peer Review

The peer review history for this article is available at https://www.webofscience.com/api/gateway/wos/peer‐review/10.1111/irv.13337.

## Supporting information


**Table S1.** Case definition for COVID‐19 and people living with HIV.
**Table S2.** International Classification of Diseases 10th Revision (ICD‐10) codes used for determining the appropriate COVID‐19 medications.
**Table S3.** International Classification of Diseases 10th Revision (ICD‐10) codes used for determining the appropriate antiretroviral therapy.
**Table S4.** International Classification of Diseases 10th Revision codes used to classify clinical severity or treatment outcomes.
**Table S5.** International Classification of Diseases 10th Revision codes used for determining the comorbidities based on the Charlson Comorbidity Index.

## Data Availability

The data supporting the findings of this study are available upon reasonable request from the corresponding author.
