# Supplementary material for: Clinical Outcomes of Coronavirus Disease 2019 in People Living With Human Immunodeficiency Virus in South Korea: A Nationwide Population‐Based Cohort Study
Source: Influenza Other Respir Viruses. 2024 Jun 10;18(6):e13337. doi: 10.1111/irv.13337 (PMC11164560; doi:10.1111/irv.13337)
Supplement: Supplementary file 1 — Table S1. Case definition for COVID‐19 and people living with HIV. Table S2. International Classification of Diseases 10th Revision (ICD‐10) codes used for determining the appropriate COVID‐19 medications. Table S3. International Classification of Diseases 10th Revision (ICD‐10) codes used for determining the appropriate antiretroviral therapy. Table S4. International Classification of Diseases 10th Revision codes used to classify clinical severity or treatment outcomes. Table S5. International Classification of Diseases 10th Revision codes used for determining the comorbidities based on the Charlson Comorbidity Index. [file IRV-18-e13337-s001.docx]

**Supplementary Materials**

**Clinical outcomes of coronavirus disease 2019 in people living with human immunodeficiency virus in South Korea: A nationwide population-based cohort study**

**Running title:** Clinical outcomes of COVID-19 in PLWH

**Table S1. Case definition for COVID-19 and people living with HIV**

| Definition | ICD-10 codes |
| --- | --- |
| COVID-19 | U071, U072, B342, andB972 |
| PLWH | B200, B201, B202, B203, B204. B205, B206, B207, B208, B209, B210, B211, B212, B213, B217, B218, B219, B220, B221, B222, B227, B230, B231, B232, B238, B24, and Z21 |

Abbreviations: ICD-10, International Classification of Diseases Tenth revision; COVID-19, coronavirus disease-2019; PLWH, people living with HIV

**Table S2. International Classification of Diseases, tenth revision (ICD-10) codes used for determining the appropriate COVID-19 medications**

| Medication | ATC | HIRA code |
| --- | --- | --- |
| Corticosteroids |  |  |
| Dexamethasone | H02AB02 | 141901ATB  142230BIJ  142232BIJ |
| Methylprednisolone | H02AB04 | 193302ATB  193305ATB  193601BIJ  193603BIJ  193604BIJ |
| Prednisolone | H02AB06 | 217001ATB |
| Hydrocortisone | H02AB09 | 170901ATB  170906ATB |
| Anticoagulants |  |  |
| Heparin | B01AB01 | 168636BIJ  168630BIJ  168632BIJ  168637BIJ  168638BIJ  168631BIJ |
| Dalteparin | B01AB04 | 140232BIJ  140230BIJ  140234BIJ  140231BIJ  140233BIJ |
| Enoxaparin | B01AB05 | 152130BIJ  152131BIJ  152132BIJ  152133BIJ  152134BIJ |
| Tocilizumab | L04AC07 | 520430BIJ  520431BIJ  520432BIJ |
| Baricitinib | L04AF02 | 667901ATB 667902ATB |

Abbreviations: ATC, Anatomical Therapeutic Chemical identifier; HIRA, Health Insurance Review and Assessment database

**Table S3. International Classification of Diseases, tenth revision (ICD-10) codes used for determining the appropriate antiretroviral therapy**

| Medication | ATC | HIRA code |
| --- | --- | --- |
| NRTI |  |  |
| Zidovudine | J05AF01 | 250201ACH |
| Lamivudine | J05AF05 | 180901ATB  180902ATB |
| Abacavir | J05AF06 | 420101ATB |
| Tenofovir disoproxil | J05AF07 | 493901ATB  664901ATB  665001ATB  665101ATB  665201ATB  665501ATB |
| Adefovir dipivoxil | J05AF08 | 457501ATB |
| Entecavir | J05AF10 | 487202ATB  487202ATD  487203ATB  487203ATD |
| Telbivudine | J05AF11 | 506001ATB |
| Clevudine | J05AF12 | 487802ACH |
| Tenofovir alafenamide | J05AF13 | 665301ATB |
| NNRTI |  |  |
| Nevirapine | J05AG01 | 200801ATB |
| Efavirenz | J05AG03 | 151002ATB |
| Etravirine | J05AG04 | 508901ATB |
| Rilpivirine | J05AG05 | 617801ATB |
| Doravirine | J05AG06 | 686101ATB |
| INSTI |  |  |
| Raltegravir | J05AJ01 | 506301ATB  506302ATB |
| Dolutegravir | J05AJ03 | 628601ATB |
| PI |  |  |
| Ritonavir | J05AE03 | 224401ATB |
| Atazanavir | J05AE08 | 458503ACH |
| Darunavir | J05AE10 | 498702ATB  498703ATB |
| Combinations |  |  |
| Zidovudine and lamivudine | JO5AR01 | 513100ATB |
| Lamivudine and abacavir | J05AR02 | 517300ATB |
| Tenofovir disoproxil and emtricitabine | JO5AR03 | 599900ATB |
| Lopinavir and ritonavir | JO5AR10 | 486300ATB |
| Lamivudine, abacavir, and dolutegravir | JO5AR13 | 641600ATB |
| Darunavir and cobicistat | J05AR14 | 647600ATB |
| Atazanavir and cobicistat | JO5AR15 | 646700ATB |
| Emtricitabine and tenofovir alafenamide | J05AR17 | 658300ATB  658400ATB |
| Emtricitabine, tenofovir alafenamide, elvitegravir, and cobicistat | JO5AR18 | 655900ATB |
| Emtricitabine, tenofovir alafenamide, and bictegravir | JO5AR20 | 676800ATB |
| Lamivudine, tenofovir disoproxil, and doravirine | J05AR24 | 686500ATB |
| Lamivudine and dolutegravir | J05AR25 | 687400ATB |

Abbreviations: ATC, Anatomical Therapeutic Chemical identifier; HIRA, Health Insurance Review and Assessment database; NRTI, nucleoside reverse transcriptase inhibitors; NNRTI, non-nucleoside reverse transcriptase inhibitor; INSTI, integrase strand transfer inhibitor; PI, protease inhibitor

**Table S4. International Classification of Diseases, tenth revision codes used to classify clinical severity or treatment outcomes**

| Clinical severity | Codes |
| --- | --- |
| ICU admission | CZ_ITEM_CD 0203 |
| Oxygen inhalation using nasal cannula or mask | M0040 |
| High flow nasal cannula | M0046 |
| Mechanical ventilation | M5850, M5857, M5858, M5859, and M5860 |
| ECMO | O1901, O1902, O1903, and O1904 |
| Vasopressor |  |
| Norepinephrine | C01CA03 |
| Epinephrine | C01CA24 |
| Vasopressin | H01BA01 |
| Dopamine | C01CA04 |
| Dobutamine | C01CA07 |
| Renal replacement therapy | O7031, O7032, O7033, O7034, O7035, O7051, O7052, O7053, O7054, and O7055 |
| Cardiac arrest | I46, M5873, M5874, M5875, M5876, M5877, and M5880 |
| Myocardial infarction | I21, I22, I252, M655x–M657x, OA631x–OA639x, OB631x–OB639x, OA641x, OA642x, OA647x, O0161x–O0171x, and O1641x–O1647x |
| Acute heart failure | I110, I130, I132, I255, I420, I425, I428, I429, I43, and I50 |

ICU, intensive care units; ECMO, extracorporeal membrane oxygenation

**Table S5. International Classification of Diseases, tenth revision codes used for determining the comorbidities based on the Charlson Comorbidity Index**

| Comorbidities | ICD-10 codes |
| --- | --- |
| Myocardial infarction | I21, I22, and I252 |
| Congestive heart failure | I099, I110, I130, I132, I255, I420, I425, I426, I427, I428, I429, I43, I50, and P290 |
| Peripheral vascular disease | I70, I71, I731, I738, I739, I771, I790, I792, K551, K558, K559, Z958, and Z959 |
| Cerebrovascular disease | G45, G46, I60, I61, I62, I63, I64, I65, I66, I67, I68, I69, and H340 |
| Dementia | F00, F01, F02, F03, G30, F051, and G311 |
| Chronic pulmonary disease | I278, I279, J40, J41, J42, J43, J44, J45, J46, J47, J60, J61, J62, J63, J64, J65, J66, J67, J684, J701, and J703 |
| Rheumatic disease | M05, M06, M315, M32, M33, M34, M351, M353, and M360 |
| Peptic ulcer disease | K25, K26, K27, and K28 |
| Mild liver disease | B18, K700, K701, K702, K703, K709, K713, K714, K715, K717, K73, K74, K760, K762, K763, K764, K768, K769, and Z944 |
| Moderate or severe liver disease | I850, I859, I864, I982, K704, K711, K721, K729, K765, K766, and K767 |
| Diabetes without complications | E100, E101, E106, E108, E109, E110, E111, E116, E118, E119, E120, E121, E126, E128, E129, E130, E131, E136, E138, E139, E140, E141, E146, E148, and E149 |
| Diabetes with complications | E102, E103, E104, E105, E107, E112, E113, E114, E115, E117, E122, E123, E124, E125, E127, E132, E133, E134, E135, E137, E142, E143, E144, E145, and E147 |
| Paraplegia and hemiplegia | G041, G114, G800, G81, G82, G830, G831, G832, G833, G834, and G839 |
| Renal disease | I120, I131, N030, N031, N032, N033, N034, N035, N036, N037, N038, N039, N050, N051, N052, N053, N054, N055, N056, N057, N058, N059, N18, N19, N250, Z490, Z491, Z492, Z940, and Z992 |
| Any malignancy | C00, C01, C02, C03, C04, C05, C06, C07, C08, C09, C10, C11, C12, C13, C14, C15, C16, C17, C18, C19, C20, C21, C22, C23, C24, C25, C26, C30, C31, C32, C33, C34, C37, C38, C39, C40, C41, C43, C45, C46, C47, C48, C49, C50, C51, C52, C53, C54, C55, C56, C57, C58, C60, C61, C62, C63, C64, C65, C66, C67, C68, C69, C70, C71, C72, C73, C74, C75, C76, C77, C78, C79, C80, C81, C82, C83, C84, C85, C88, C90, C91, C92, C93, C94, C95, C96, and C97 |
| Organ transplantation | Z94 |
